# Hereditary hemorrhagic telangiectasia: from signaling insights to therapeutic advances

**DOI:** 10.1172/JCI176379

**Published:** 2024-02-15

**Authors:** Tala Al Tabosh, Mohammad Al Tarrass, Laura Tourvieilhe, Alexandre Guilhem, Sophie Dupuis-Girod, Sabine Bailly

**Affiliations:** 1 Biosanté Unit U1292, Grenoble Alpes University, INSERM, CEA, Grenoble, France.; 2 Hospices Civils de Lyon, National HHT Reference Center and Genetics Department, Femme-Mère-Enfants Hospital, Bron, France.; 3 TAI-IT Autoimmunité Unit RIGHT-UMR1098, Burgundy University, INSERM, EFS-BFC, Besancon, France.

## Abstract

Hereditary hemorrhagic telangiectsia (HHT) is an inherited vascular disorder with highly variable expressivity, affecting up to 1 in 5,000 individuals. This disease is characterized by small arteriovenous malformations (AVMs) in mucocutaneous areas (telangiectases) and larger visceral AVMs in the lungs, liver, and brain. HHT is caused by loss-of-function mutations in the BMP9-10/ENG/ALK1/SMAD4 signaling pathway. This Review presents up-to-date insights on this mutated signaling pathway and its crosstalk with proangiogenic pathways, in particular the VEGF pathway, that has allowed the repurposing of new drugs for HHT treatment. However, despite the substantial benefits of these new treatments in terms of alleviating symptom severity, this not-so-uncommon bleeding disorder still currently lacks any FDA- or European Medicines Agency–approved (EMA-approved) therapies.

## Genetic basis of HHT

Hereditary hemorrhagic telangiectasia (HHT) or Osler-Weber-Rendu syndrome is a hereditary disease that is transmitted in an autosomal dominant manner and is caused by loss-of-function (LOF) mutations in certain components of the predominantly endothelial BMP9-10/ENG/ALK1/SMAD4 signaling pathway, which is an important mediator of vascular quiescence (1). Specifically, mutations in *ENG* (chromosomal locus 9q34.11, encoding the coreceptor endoglin) and *ACVRL1* (chromosomal locus 12q13.13, encoding the type I receptor ALK1) are nearly equally responsible for the majority of HHT cases and give rise to the two major forms of the disease, HHT1 (OMIM 187300) and HHT2 (OMIM 600376), respectively (2–4). Collectively, over 600 different HHT-associated pathogenic mutations in *ENG* and *ACVRL1,* spanning the entire coding sequences of both genes, have been documented in the ClinVar database (5). While reported *ENG* mutations were mostly nonsense or frameshift mutations leading to premature termination codons, a higher prevalence of missense mutations was observed for *ACVR*L1. Mutations in the *SMAD4* gene (chromosomal locus 18q21.2) were also found in HHT patients, although more rarely than *ENG* and *ACVRL1* mutations, resulting in a combined syndrome of HHT and juvenile polyposis (JP-HHT; OMIM 175050) (4, 6). Based on the *SMAD4* mutation repository developed by Wooderchak et al. (7), at least 26 different mutations in *SMAD4* have been described in patients with the combined JP-HHT syndrome, most of which are missense mutations and deletions that are mainly concentrated at the MH2 domain of SMAD4.

Together, mutations in *ENG*, *ACVRL1*, and *SMAD4* are responsible for more than 90% of HHT cases, leaving a minority of clinically diagnosed individuals with an unknown genetic basis. In some cases, this can be attributed to limitations of current sequencing technologies in detecting certain variants in the previously identified loci or to the exclusion of testing noncoding regions (8); however, additional chromosomal loci could also be implicated in HHT development. As such, mutations in *GDF2* (chromosomal locus 10q11.22, encoding BMP9) were described in few cases displaying an HHT-like syndrome (9–13). This rare form of HHT was annotated as HHT5 (OMIM #615506). In addition, mutations in *RASA1*, which are commonly associated with hereditary capillary malformations with or without arteriovenous malformations (AVMs) (14, 15), have been reported in few patients presenting HHT symptoms (16–18). More recently, LOF variants in *EPHB4* (encoding ephrin receptor B4) were reported in a few individuals exhibiting atypical HHT symptoms and HHT-like hepatic abnormalities (19). Furthermore, one study described an overrepresentation of rare mutations in *DROSHA*, a key enzyme involved in miRNA processing, among clinically diagnosed HHT patients who did not carry any mutations in the typical HHT-associated genes (20). Interestingly, this study also demonstrated that zebrafish and mice with endothelial-specific DROSHA deficiency developed vascular defects resembling those observed in HHT patients (20).

## Clinical manifestations of HHT

**Figure 1:**
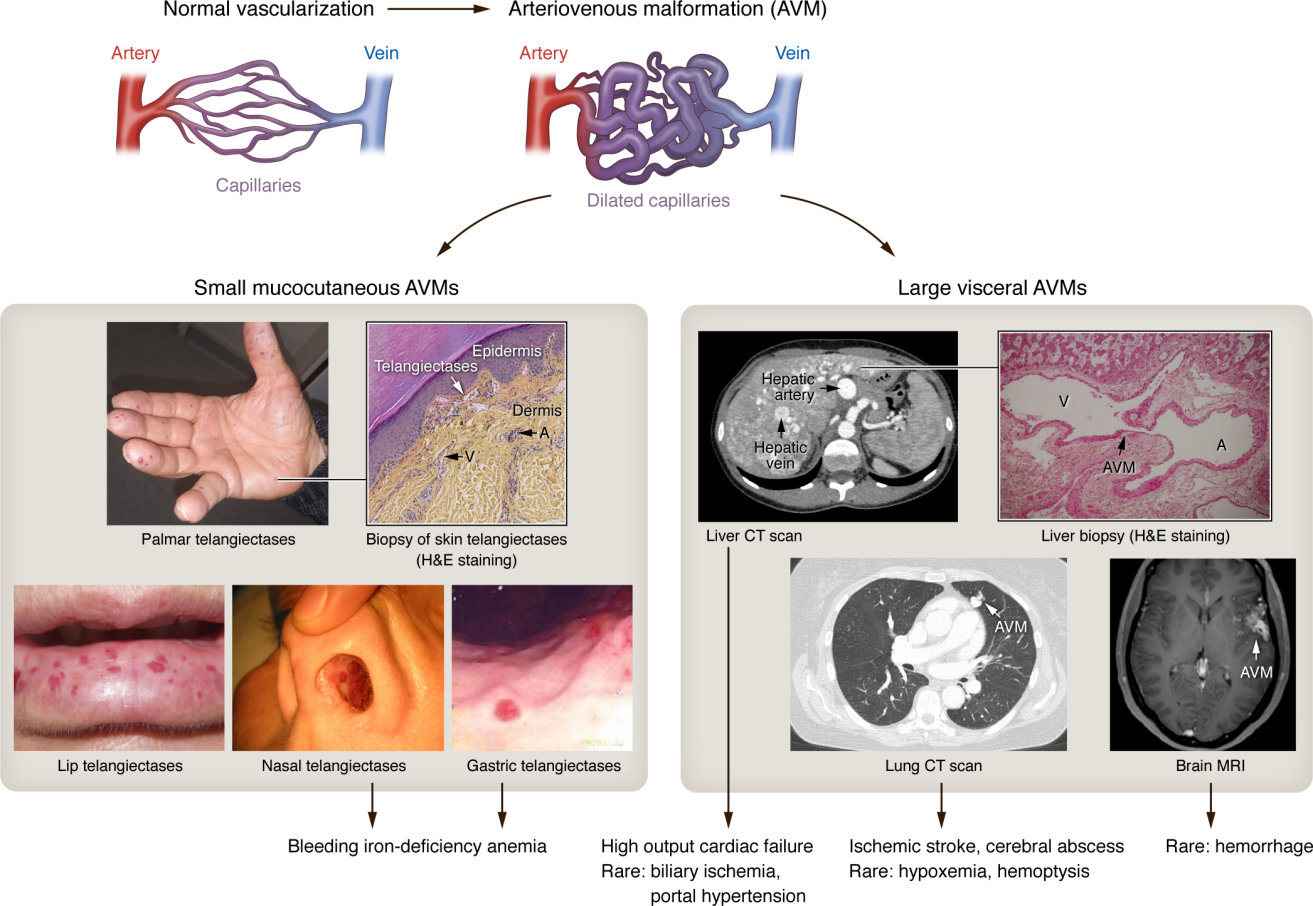
AVMs: The hallmark of HHT and its clinical consequences. In HHT, focal dilations of the postcapillary venules (indicated on images by V) progressively encompass the normal capillaries and establish a direct connection with dilated arterioles (indicated by A), leading to AVM formation, muscularization of large vessels, and dilation of capillary beds, which are often surrounded by inflammatory cells. At the microvascular level, AVMs appear as telangiectases on specific mucocutaneous areas (finger pads, lips, tongue, nasal and digestive mucosa). They are responsible for spontaneous and recurrent epistaxis/bleeding, leading to bleeding iron-deficiency anemia. At the macrovascular level, large AVMs mainly affect the liver, leading to high-output cardiac failure, and more rarely biliary ischemia and portal hypertension; lung AVMs can provoke ischemic stroke and cerebral abscess and, more rarely, hypoxemia and hemoptysis. Central nervous system AVM is rarely complicated by hemorrhage. Skin and liver sections were stained for H&E. Photo of the liver section was provided by J.Y. Scoazec (Institut Gustave Roussy, University Paris-Saclay, Paris, France).

AVMs are the hallmark of HHT. According to the international classification of vascular anomalies, AVMs are characterized by malformed arteries, veins, and capillaries with direct arteriovenous communications resulting in arteriovenous shunting (21) (Figure 1). The stepwise development of AVMs in the context of HHT was histologically documented in cutaneous biopsies by Braverman et al. in 1990 (22). This process was found to commence with focal dilatations of the postcapillary venules that progressively encompass the normal capillaries, finally establishing a direct connection with dilated arterioles. The venous origin of AVMs was later supported by studies specifically deleting *Alk1* or *Eng* from venous and capillary beds, which was sufficient to obtain AVMs in retinas (23, 24). In addition, the process seemed to involve an immune arm, as perivascular lymphocytic infiltrates were evident at the site of the AVM (22). It is noteworthy that all clinical signs of HHT result from AVMs of varying size and number affecting different organ systems.

At the mucocutaneous surfaces, HHT AVMs appear as telangiectases, after which the disease is named. HHT-associated telangiectases are typically small, red, flat, or slightly elevated spots that blanch under pressure. They are classically present on the palmar faces of finger pads and hands, the lips, the tongue, and mucosal areas inside of the mouth, the nasal cavity, and the gastrointestinal (GI) tract (25, 26) (Figure 1). On the nasal mucosa, telangiectases are responsible for the spontaneous and recurrent epistaxis leading to iron-deficiency anemia in about 50% of patients (27). Similarly, GI telangiectases, which can affect any part of the tract, occasionally result in occult bleeding.

In visceral organs, AVMs can reach a larger size in the lungs, the central nervous system, and the liver and result in life-threatening complications (Figure 1). Pulmonary AVMs can cause direct complications, such as hemorrhage/hemoptysis, due to rupture and hypoxemia due to shunting, but can also cause indirect complications by allowing systemic embolic events resulting in brain abscesses and embolic strokes (28). On the other hand, hepatic AVMs can lead to high-output cardiac failure, biliary ischemia, and portal hypertension (29), warranting liver transplantation. Interestingly, hepatic relapse of the vascular lesions has been described in some HHT patients receiving liver transplants (30), suggesting a potential role of circulating endothelial precursors, which bear the HHT-causal mutation, in populating the newly transplanted liver and driving the formation of new vascular lesions. Cerebral and spinal AVMs are less frequent and generally asymptomatic. Although they can cause serious complications, such as hemorrhagic stroke or epilepsy, the risks associated with treatment are currently higher than those of natural progression and unruptured cerebral AVMs are generally not treated. Widespread screening of asymptomatic HHT patients is therefore still controversial (31).

Cohort studies have demonstrated interorgan differences in the natural history of AVMs. Mucocutaneous telangiectases and liver AVMs appear progressively over the life span, with complications generally occurring in late adulthood. The majority of lung AVMs are probably present from childhood, but some of them become detectable only during adulthood. Most brain AVMs appear in utero or in early childhood and generally remain stable later in life (32). Although HHT1 and HHT2 patients are clinically indistinguishable, *ACVRL1* mutations are associated with higher rates of liver AVMs and digestive telangiectases, while *ENG* mutations are more predominantly linked to pulmonary and cerebral AVMs (33).

In addition to the well-documented HHT symptoms, i.e., epistaxis, telangiectases, and AVMs, HHT patients exhibit a heightened infectious risk in soft tissues, bones, and joints involving *Staphylococcus aureus* as well as cerebral infections involving bacteria from the orodigestive flora (34). HHT is also associated with immunological abnormalities, mainly characterized by a T-helper lymphopenia, although these abnormalities lack clear correlation with the aforementioned infectious risks (35).

## The second-hit hypothesis

The LOF nature of HHT causal mutations and the autosomal dominant inheritance of the disease led to the longstanding belief that HHT is caused by haploinsufficiency of the mutated gene product. This was corroborated by several reports demonstrating a reduction in endoglin levels in HHT1-derived cells (36–38) and in ALK1 levels in some HHT2-derived cells compared with control counterparts (37–39). In line with these studies, mice heterozygous for mutations in *Eng* or *Acvrl1* (*Eng^+/–^* or *Alk1^+/–^*) display reduced expression levels of the affected gene and develop some HHT-like lesions at the adult stage, albeit with a low penetrance, including telangiectases, nosebleeds, and dilated vessels with reduced vascular smooth muscle cell (VSMC) coverage (40–42).

However, the haploinsufficiency model does not explain why HHT vascular lesions develop focally in characteristic vascular beds despite the presence of the germline HHT causal mutation in all endothelial cells (ECs) of the body (43). This model also fails to elucidate the differences in disease expressivity that are observed even between related patients carrying the same mutation (44). These disparities put forth the second-hit hypothesis in HHT, which is becoming more and more accepted by the HHT scientific community (45). This hypothesis implies that the germline mutation (first hit) predisposes the endothelium to vascular defects that strictly develop in the presence of additional, local factors (second hit) that could be either environmental or genetic. Along these lines, several preclinical studies have demonstrated the role of proangiogenic and proinflammatory triggers in driving HHT pathogenesis (46). For instance, *Eng^+/–^* or *Alk1^+/–^* mice acquired cerebrovascular dysplasia 8 to 10 weeks after intracranial administration of viral vectors encoding VEGF, as opposed to normal angiogenesis in WT mice receiving the same treatment (47, 48). In addition, unlike WT mice, inducible *Eng*- or *Alk1-*knockout mice readily and consistently developed AVMs in their brains only upon local VEGF overexpression (49–52) as well as dermal AVMs strictly after wounding or upon local VEGF or LPS treatment (53–56). Interestingly, blocking VEGF in inducible *Alk1*^–/–^ mice partially reversed or blocked the development of AVMs induced by VEGF, LPS, or wounding (51, 55), in line with the well-documented effectiveness of the humanized anti-VEGF antibody bevacizumab in alleviating HHT symptoms (Table 1) (57, 58). In addition to angiogenic and inflammatory stimuli, several other environmental triggers have been proposed as potential second hits in HHT, including mechanical stress and sun exposure. A study on 103 HHT patients revealed a higher number of telangiectases on the dominant hand and on the lower lip, which are expected to be more frequently exposed to mechanical stimuli, than the other hand and the upper lip (59). The number of telangiectases on the lips was also found to be positively correlated with sunlight exposure (59).

Another study supporting the two-hit hypothesis in HHT patients was released in 2019, but this time involving a genetic second hit (60). Using next-generation sequencing, Snellings et al. detected low-frequency somatic mutations, mostly in trans configuration, leading to biallelic loss of *ENG* or *ACVRL1* in cutaneous telangiectases on the hands of some HHT patients (60). Consequently, HHT vascular malformations were proposed to develop when specific endothelial clones acquire somatic mutations leading to loss of heterozygosity (60). This is a well-known pathogenic mechanism in cancer, known as the Knudsonian two-hit mechanism (61), that was also shown in some heritable vascular anomalies, including venous, glomuvenous, and cerebral cavernous malformations (62, 63). It remains to be validated whether this hypothesis holds true for HHT-related AVMs. As sunlight comprises mutagenic ultraviolet radiation, it is plausible that prolonged exposure to sunlight may trigger somatic mutations that potentially support the development of dermal telangiectases on the hands. This hypothesis might explain the late-onset development of some AVMs (skin, liver) (32), but cannot explain the ones that appear early in life (cerebral and pulmonary AVMs) (32, 64). Therefore, the somatic second-hit hypothesis could indeed be responsible for some HHT-related vascular defects, but might not represent a universal pathogenic mechanism in HHT.

## Signaling pathways involved in HHT

### BMP9-10/ENG/ALK1/SMAD4 pathway.

**Figure 2:**
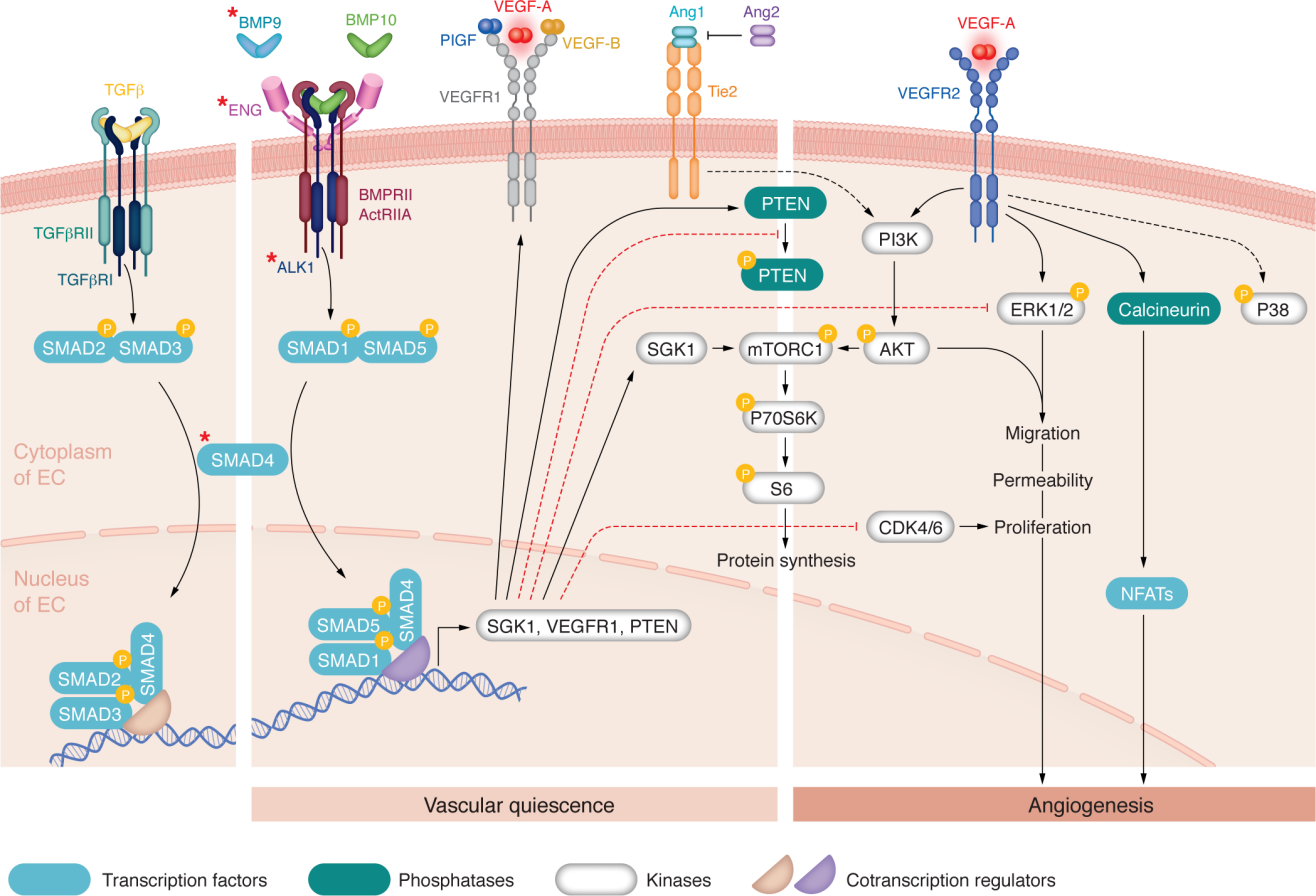
BMP9-10/ENG/ALK1/SMAD4 signaling pathway maintains vascular quiescence by repressing angiogenic pathways. HHT occurs due to LOF mutations in *ENG*, *ALK1*, *SMAD4*, and, more rarely, *BMP9* (respective proteins indicated with red asterisks), which are all in the same signaling pathway. On endothelial cells, BMP9 or BMP10 recruits a heterocomplex composed of two type II receptors (BMPRII or ActRIIA, which are the main type II receptors expressed on ECs, and two similar type I receptors (ALK1), and the coreceptor ENG (endoglin). Upon BMP binding, the type II receptor phosphorylates ALK1, which subsequently phosphorylates the transcription factors SMAD1/5. SMAD1/5 bind SMAD4, which is shared with the TGF-β pathway, to regulate transcription of many genes (in association with other transcription factors). BMP9 and BMP10 maintain vascular quiescence (middle panel) via repression of angiogenesis pathways (right panel). VEGF-A (red) binds to VEGFR2, which activates the ERK1/2 and P38 MAPK pathways and the PI3K/AKT/mTORC1 pathway. In turn, the PI3K/AKT/mTORC1 pathway activates the signaling cascade P70S6K/S6. VEGF can also activate the calcineurin phosphatase, which activates, via dephosphorylation, the NFAT transcription factor family. The PI3K/AKT/mTOR pathway is negatively regulated by the phosphatase PTEN, which is active when unphosphorylated. VEGF-A can also bind to VEGFR1, but this will not generate a signal. Two other members of the VEGF family, VEGF-B (yellow) and PlGF (blue), also bind to VEGFR1. BMP9 induces the expression of VEGFR1, thus inhibiting VEGF signaling. BMP9 also induces PTEN expression and phosphorylation, which inhibit AKT activity as well as the expression of SGK1 kinase, which can activate the mTORC1/P70S6K/S6 pathway. Moreover, BMP9 inhibits ERK activation and CDK4/6 kinases through not-yet-characterized mechanisms. Ang1 activates the TIE2 receptor to maintain vascular quiescence, and this pathway can be antagonized by Ang2.

Endoglin and ALK1 are two transmembrane receptors mainly expressed on ECs, explaining why mutations in this pathway result in vascular abnormalities. BMP9 and BMP10 are the high-affinity ligands of the receptors ALK1 and endoglin (65, 66). Endoglin is a coreceptor for BMP9 and BMP10 and serves as a reservoir of these ligands on the surface of ECs (67), enhancing ligand-induced responses (65). In line wit endoglin’s role as an upstream coreceptor of the ALK1 pathway, it was shown that AVMs induced by endothelial loss of *Eng* could be corrected by overexpression of ALK1, whereas endoglin overexpression could not compensate for the loss of *Alk1* (68). BMP9 or BMP10 recruits a heterocomplex of two type II receptors (BMPRII or ActRIIA, which are the main type II receptors expressed on ECs) and two type I receptors (ALK1) (1, 69). Upon BMP9 or BMP10 binding, the serine/threonine kinase type II receptor phosphorylates the serine/threonine kinase type I receptor ALK1, leading to its activation. Subsequently, activated ALK1 phosphorylates the transcription factors SMAD1/5, allowing their binding to SMAD4, which is a common downstream signaling mediator shared with the TGF-β pathway (Figure 2). The trimeric SMAD complex then migrates to the nucleus, where it interacts with other transcription factors to regulate the transcription of many target genes (1, 69). Accordingly, endothelium-specific *Smad1/5* or *Smad4* deletions resulted in AVM formation in the retina (70–72). In addition, few studies show that BMP9 and BMP10 can activate noncanonical BMP signaling pathways, including P38, ERK, Wingless (Wnt), and NOTCH signaling (73). It is widely accepted that BMP9 and BMP10 lead to vascular maturation and quiescence (74). The current working model suggests that BMP9-10/ENG/ALK1/SMAD4 signaling maintains vascular homeostasis via attenuation of proangiogenic pathways and, in particular, the VEGF signaling pathway. However, the mechanisms underlying this attenuation are still not fully characterized.

**Figure 3:**
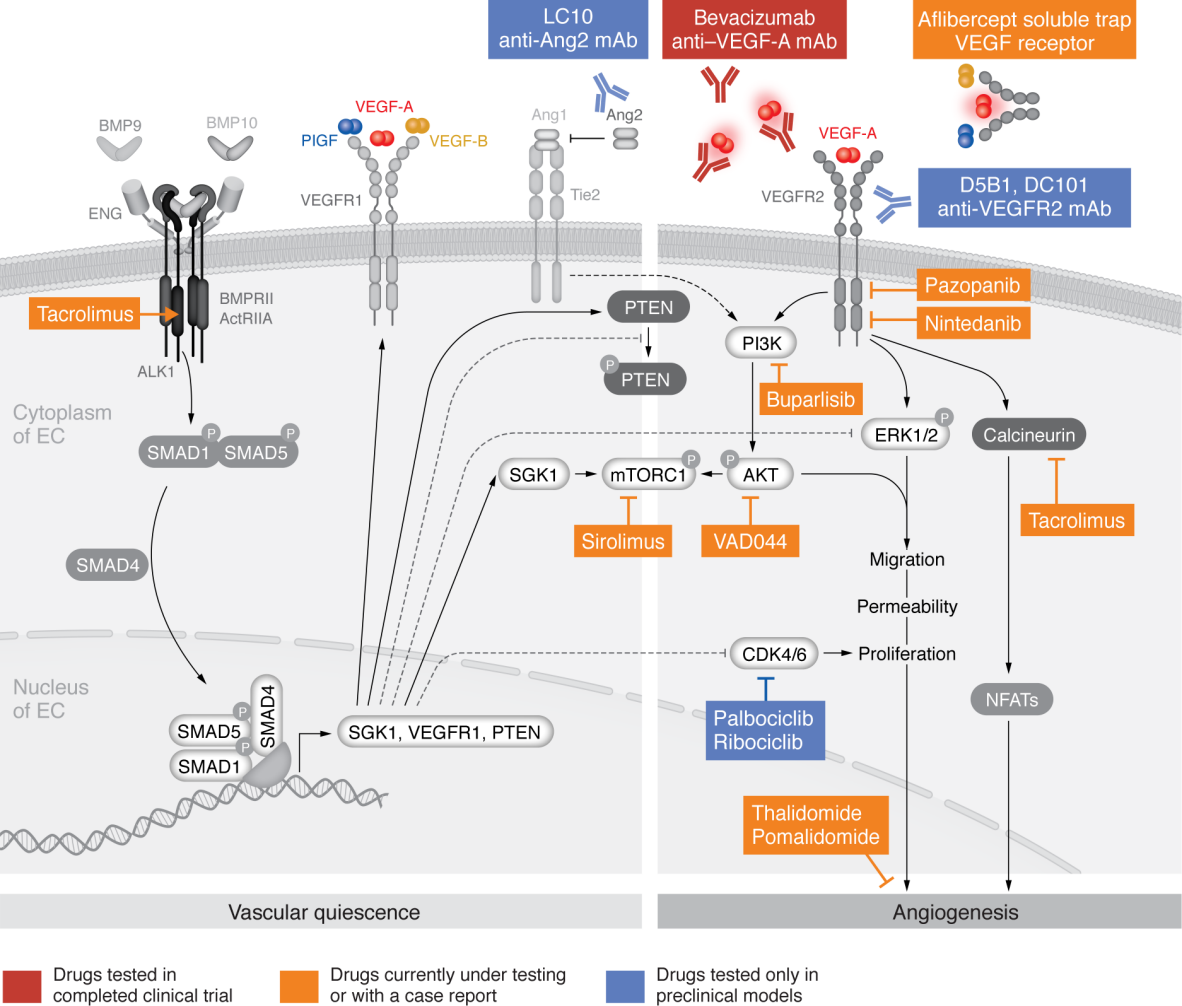
Therapeutic targets of antiangiogenic drugs tested in preclinical models and in HHT patients. Figure shows the targets of drugs tested in completed clinical trials (red), drugs currently under testing or with a case report (orange), and drugs tested only in preclinical models (blue). For further details, see Table 1 and Table 2. Drugs have been developed to block VEGF-A signaling using neutralizing anti–VEGF-A mAbs (bevacizumab) or soluble trap/decoy receptors that bind VEGF-A (red), VEGF-B (yellow), and PlGF (blue) (aflibercept), or neutralizing anti-VEGFR2 antibodies (D5B1 and DC101). Drugs developed to block intracellular signaling, such as tyrosine kinase receptor inhibitors that block VEGFR2 activity but also other receptors, are currently undergoing testing: pazopanib (VEGFR, PDGFR, c-KIT, and FGFR) and nintedanib (VEGFR, PDGFR, and FGFR). Drugs have also been developed to block PI3K and AKT (VAD044), as well as mTORC1 (sirolimus) and calcineurin and FKBP12 (tacrolimus). Immunomodulatory imide drugs (IMIDS), such as thalidomide and pomalidomide, have been tested in HHT patients. Other drugs have been tested so far only in preclinical models, such as the neutralizing anti-Ang2 monoclonal antibodies (LC10) and inhibitors of CDK4/6 (palbociclib and ribociclib).

The immunosuppressor tacrolimus (FK506) has been identified in two independent screens as a potent activator of SMAD1/5 signaling using a reporter assay based on the Id1 promoter (75, 76). However, the mechanism by which tacrolimus activates this pathway is not clearly understood. Tacrolimus is a macrolide with immunomodulatory and antiangiogenic properties commonly used in patients who have undergone organ transplantation (77). It inhibits the phosphatase calcineurin, which dephosphorylates NFAT proteins (Figure 3) (78, 79). Activation of calcineurin has also been shown to be downstream of VEGF (80) (Figure 2). Interestingly, we have previously shown that BMP9 regulates the calcineurin/NFAT pathway in lymphatic differentiation (81). Tacrolimus can also activate the BMP signaling pathway by blocking 12 kDa FK506 (FKBP12) (Figure 3) (82), which is known to bind and suppress ALK1 activation (76). In ECs, it was shown that tacrolimus activated SMAD1/5 signaling and inhibited AKT and P38 phosphorylation induced by VEGF (75). The same group showed that tacrolimus injection in BMP9/BMP10-immunodepleted postnatal retinas prevented hypervascularization (Table 2). However, the molecular mechanisms involved in this protective effect of tacrolimus are not yet fully elucidated.

### VEGF/VEGFR2 pathway.

VEGFs, which include VEGF-A, VEGF-B, and PlGF (placenta growth factor), are some of the most potent and extensively studied angiogenic factors. VEGF signaling occurs through its binding to the receptor tyrosine kinase VEGFR2, which activates several downstream pathways (Figure 2). These include the ERK1/2 pathway, the PI3K/AKT/mTOR pathway, the SRC and small GTPases pathways, and others that are poorly understood, including the p38 MAPK pathway (80, 83) (Figure 2). The PI3K/AKT/mTOR pathway is negatively regulated by the phosphatase and tensin homolog (PTEN), which is active when unphosphorylated. VEGF-A, VEGF-B, and PlGF can also bind VEGFR1 with higher affinity than VEGFR2, but the former exhibits low kinase activity, making VEGFR1 a decoy receptor (80). VEGF signaling is thus modulated by the different relative expression levels of VEGFR2 versus VEGFR1 in ECs.

Awareness of the beneficial effect of blocking VEGF signaling in HHT patients dates back to 2006, when an HHT patient suffering from a malignant mesothelioma unexpectedly showed amelioration of HHT symptoms following antiangiogenic cancer treatment with an anti-VEGF-A monoclonal antibody (bevacizumab) (84). The following year, BMP9 and BMP10 were identified as two high-affinity ligands for the receptors ALK1 and endoglin (65, 66). It was shown that these two ligands inhibited angiogenesis in vitro (EC proliferation and migration) and in ex vivo models (66, 85). A few years later, the first clinical trial using bevacizumab showed very positive results on 25 patients suffering from HHT (Table 1) (86). Since then, preclinical HHT models have been developed in order to test different therapeutic approaches. The main model used is the murine retina, a two-dimensional–like vascular structure that forms via angiogenesis during early postnatal days. It has been shown that the loss of *Alk1*, *Eng*, *Smad1/5*, *Smad4*, or *Bmp9/Bmp10* led to spontaneous AVMs in the retina (46). Using these models, it was shown that blocking VEGF signaling reduced the development of vascular malformations (51, 55). These mouse models supported that AVM formation involved aberrant EC responses, including enhanced proliferation, impaired flow-migration coupling, and abnormal cellular behavior in response to angiogenic signals such as VEGF (46).

To date, there is a limited understanding of how BMP9 or BMP10 mitigates VEGF signaling. It has been found that BMP9 induces the expression of VEGFR1, thus restricting downstream VEGF signaling (87) (Figure 2). Conversely, inhibiting ALK1 signaling in ECs using ALK1 ligand trap (ALK1-Fc) promoted an elevation of several key proangiogenic regulators (*DLL4*, *ANGPT2* [encoding angiopoietin 2], and *KDR* [encoding VEGFR2]) (75). Accordingly, it was shown that *Alk1*^+/–^ mice presented a reduced level of VEGFR1 expression and that VEGFR1 levels were reduced in skin biopsies from HHT2 patients (88).

Concerning the molecular mechanisms involved downstream of the VEGF signaling pathway, studies in mice have shown that the loss of *Alk1* or *Smad4* resulted in the activation of the PI3K/AKT pathway (70, 89, 90). Similar results were obtained in vitro in HUVECs using siRNA against ALK1 (70, 90). Inversely, BMP9 treatment for 2 hours in HUVECs was found to increase PTEN expression and activity, leading to a decrease in AKT activity (Figure 2) (89, 90). Activated AKT subsequently activates the mTORC1 complex, which in turn activates the signaling cascade P70S6K/S6 (Figure 2). Interestingly, this pathway has been found activated in skin telangiectases from HHT patients (89, 91). In HUVECs, BMP9 was also found to induce the expression of SGK1 kinase, which can also activate the mTORC1/P70S6K/S6 pathway (92). In this work, it was proposed that activation of this pathway would play a role in regulating protein synthesis. Additionally, BMP9 was also shown to inhibit ERK activation, although the specific mechanism behind this inhibition remains unclear (Figure 2). In parallel, BMP9 and BMP10 have been shown to inhibit endothelial cell proliferation, but the underlying mechanism is not yet clearly characterized. One study showed that BMP9-induced inhibition of EC proliferation was SMAD1/5 dependent and required the expression of the CDK4/6 inhibitor P27^KIP^ (93). In contrast, *Smad4* loss enhanced flow-mediated KLF4/TIE2/PI3K/AKT signaling, leading to cell-cycle progression, which was inhibited using CDK4/6 inhibitors (94). Accordingly, the CDK4/6 inhibitors and ribociclib inhibited the formation of retinal AVMs and the knockdown of CDK6 prevented the development of retinal AVMs (Table 2) (94, 95).

### Angiopoietin/Tie2 pathway.

Genetic HHT mouse models have also shown an increase in Angiopoietin 2 (Ang2) levels (96, 97). The angiopoietin/Tie2 pathway is critical for maintaining vessel stability by regulating, as for VEGF, the PI3K/AKT pathway (Figure 2). Angiopoietin 1 (Ang1), produced by mural cells, activates the receptor tyrosine kinase TIE2 to maintain VSMC coverage. An increase in TIE2 antagonist Ang2 produced by ECs inhibits Ang1-mediated TIE2 activation, leading to destabilization of VSMC-EC interaction and a decrease in vascular quiescence (83, 98). Accordingly, it was recently shown that blocking Ang2 (using LC10 monoclonal antibody) can prevent or reduce AVMs and other HHT-associated abnormalities in mice (96, 97) (Figure 3). However, in contrast with these mouse models, Ang2 circulating levels in HHT patients tend to be reduced compared with those in healthy individuals (99, 100). Thus, although Ang2 seems to be a promising new therapeutic target, further work is needed before pursuing clinical trials (101).

## Preclinical data, clinical trials, and perspectives in HHT

Since 2009, antiangiogenic drugs developed in oncology have been increasingly used in drug-repurposing strategies in patients with HHT (102, 103). Today, numerous preclinical (51, 55, 70, 75, 88, 90, 94–97, 104–112) and clinical studies (58, 86, 104, 113–136) targeting angiogenesis in the scope of HHT have been carried out and are summarized in Table 1 and Table 2. Drugs used in the aforementioned studies and their distinct sites of action are depicted in Figure 3.

Today, with our current knowledge of the pathophysiological mechanisms of HHT owing to preclinical models and clinical trials, the main therapeutic lines of action to be considered include the following: first, avoiding angiogenic and inflammatory stimuli by all means, in line with the second hit hypothesis. Whenever possible, “mechanical” prevention, such as sun protection, application of nasal ointments, and avoidance of nasal cauterization during childhood, is recommended to reduce the development of cutaneous and mucosal telangiectases. In addition, it is essential to prevent and treat anemia and iron deficiency, whose repercussions are probably not limited to hematology alone, but also include the risk of infection and stroke (43, 137).

Another important point is moving toward personalized medicine for each patient. While studies across 14 years that involved more than 400 patients ensured the safety of bevacizumab, they demonstrated that the response varied depending on its elimination rate (58, 86). Indeed, pharmacokinetic studies highlighted a correlation between a progressive decrease in response to the drug and low plasma bevacizumab concentration. Thus, as in cancer, there is a concentration threshold for efficacy. So taking into account the metabolisms of different patients and adapting the dosage accordingly would allow us to maintain its efficacy and optimize the risk-benefit balance. It is therefore essential to monitor clinical improvement as a function of bevacizumab levels during maintenance therapy in order to maintain bioactive levels within the therapeutic window, especially if efficacy appears to be declining (120). In case of treatment failure, it is mandatory to verify that bevacizumab residual levels are above 25 mg/L (120, 138).

Another aspect that should be considered more carefully is tailoring treatment to disease severity in order to limit drug toxicity. To reduce systemic adverse effects, antiangiogenic treatments applied locally have been studied. For example, 0.1% tacrolimus ointment applied twice daily has promising outcomes (139) and could be a good option for patients with moderate epistaxis, although this awaits further confirmation by larger studies. Moreover, continued use of tacrolimus in HHT patients could be called into question, as a few HHT patients using this regimen after liver transplantation developed new hepatic vascular lesions and needed a second transplantation (30). Other antiangiogenic drugs with local use are more debated. Two randomized trials showed that intranasal bevacizumab was not effective (140, 141). This result was confirmed in a systematic review (142) conducted on 13 studies. Some studies evaluated submucosal administration alone (143, 144), with electrocautery (145) or cyanoacrylate glue (146). In addition, nonselective β-adrenergic receptor blockers with antiangiogenic properties that reduce VEGF and matrix metalloproteinase-9 (MMP-9) tissue expression have been studied (147). For instance, four randomized studies using topical timolol in the form of a nasal spray or gel for epistaxis and an ophthalmic solution for cutaneous telangiectases reported discordant results in cohorts ranging from 6 to 58 patients (148–151). This is consistent with a systematic review from 2022 (152) on topical beta blockers suggesting that propranolol is more promising than timolol. Topical propranolol was reported to increase hemoglobin levels (153, 154), but its use warrants monitoring for bradycardia occurrence, even when administered locally.

## Future challenges in HHT

Although great progress has been made in the treatment of HHT patients, there are still some remaining challenges. Perhaps the greatest challenge is determining whether large AVMs observed in HHT patients would respond to antiangiogenic therapies. Indeed, several antiangiogenic drugs, particularly those administered orally, including tyrosine kinase inhibitors, AKT inhibitors, pomalidomide, and mTOR inhibitors, are currently being tested on visceral AVMs, but no regression of visceral AVMs has been observed so far. Only skin or mucosal telangiectases have been found to be reduced in a few cases (125, 128). In the Metafore study (86), cardiac index and hepatic blood flow were decreased after bevacizumab treatment, but large liver AVMs on CT scan were unchanged, and despite a marked improvement in digestive bleeding, the number of GI telangiectases was not reduced. Although some preclinical studies reported that antiangiogenic treatments, such as PI3K inhibitors, could revert established AVMs in preclinical models (90), these AVMs were only seen in neonatal retinas of mice. Similarly, it was shown that in zebrafish *Eng* mutants, the HHT-like phenotype was abrogated by inhibiting VEGF signaling with drugs targeting VEGFR2, but only in embryos (112). In both models, the treatment was effective during active angiogenesis (embryonic or postnatal stages), which is not the case after remodeling of vascular structure in large AVMs. Thus there is a real need for testing the efficacy of drugs in preclinical models on large AVMs or AVMs at adult stages. Accordingly, recent preclinical HHT models that better mimic HHT symptoms have been developed in adult mice and zebrafish (55, 106, 107, 112). However, to maximize the potential of currently available models for monitoring the response of AVMs to tested drugs, advancements in visualization methodologies are required for the deeper investigation of AVM formation, progression, and regression. Such advancements might also allow the revelation of previously undetected visceral AVMs in HHT mouse models, necessitating further investigation of these models using advanced visualization tools in the future.

Another challenge is to identify the best target to inhibit angiogenesis in HHT patients. VEGF signaling is complex and drives numerous downstream pathways, so adverse complications can be caused by inhibiting the VEGF pathway as a whole (155). Thus, one could imagine that targeting specific downstream pathways of VEGF could reduce these complications. A recent study addressed this point by using an adult endoglin-mutant zebrafish model that developed several HHT symptoms (112). They found that inhibiting mTORC1 (using rapamycin, also known as sirolimus) or MEK pathways prevented vascular abnormalities (Table 2), whereas inhibiting P38 or nitric oxide synthase pathways had no effect. Combined subtherapeutic mTORC1 and MEK inhibitors demonstrated synergistic effects in treating HHT. However, it remains unclear at this stage whether it will be more beneficial in the future to only target particular downstream targets of VEGF (PI3K, MEK), which are activated by many signaling pathways, or to target a specific proangiogenic growth factor, such as VEGF.

In the present Review, we focus on therapeutic approaches that aim to block activated signaling pathways due to LOF of the BMP9-10/ALK1 signaling pathway, but another possibility would be to increase the deficient signaling pathway (more ligands, more receptors, activation of the downstream signaling pathway). However, the validity of such a therapeutic option fundamentally relies on whether HHT vascular lesions are driven by haploinsufficiency or biallelic loss of the affected gene.

## Summary and conclusions

Ever since the discovery that HHT is due to mutations in a single signaling pathway nearly 30 years ago, considerable progress has been made in treating HHT symptoms by blocking the VEGF angiogenic pathway. However, the molecular mechanism underlying the interaction between BMP9/BMP10 and VEGF signaling is still not fully elucidated. In addition, as described in this Review, several points remain to be better characterized, such as the development of AVMs, preclinical adult models for AVMs, biomarkers for personalized antiangiogenic treatments, the choice of therapeutic target (growth factors such as VEGF or more focused downstream signaling [AKT, MTORC1, MEK]), the possibility and efficacy of localized treatment and the benefit of preventative antiangiogenic treatments. Larger scale and real-life data are particularly difficult to obtain, since these treatments are used off-label due to a lack of better options. This is partly due to the general abstention of pharmaceutical industries from repurposing drugs in rare diseases. In the meantime, antiangiogenic drugs hold a promising potential in HHT and represent avenues worth investigating before taking on the future challenges associated with gene therapy.

## Figures and Tables

**Table 2:**
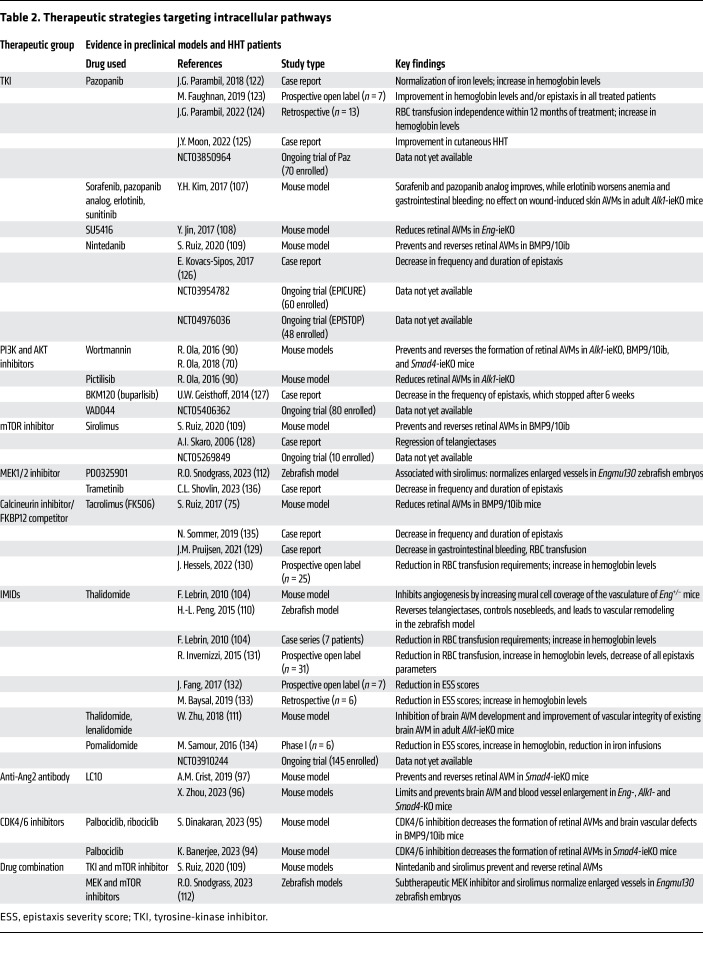
Therapeutic strategies targeting intracellular pathways

**Table 1:**
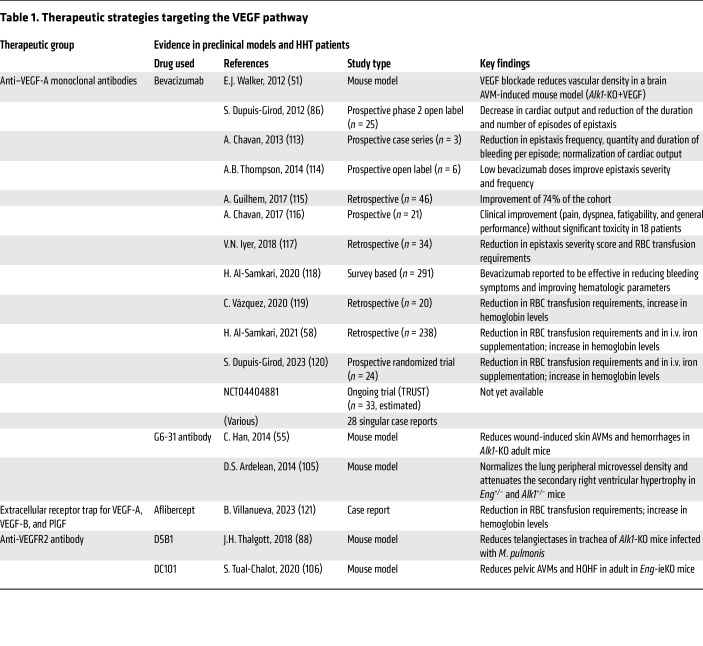
Therapeutic strategies targeting the VEGF pathway

## Version 1 02/15/2024

Electronic publication
